# Post-Traumatic Stress Disorder and severe maternal morbidity: is there an association?

**DOI:** 10.6061/clinics/2018/e309

**Published:** 2018-04-17

**Authors:** Carina R. Angelini, Rodolfo C. Pacagnella, Mary A. Parpinelli, Carla Silveira, Carla B. Andreucci, Elton C. Ferreira, Juliana P. Santos, Dulce M. Zanardi, Renato T. Souza, Jose G. Cecatti

**Affiliations:** I Departamento de Ginecologia e Obstetricia, Faculdade de Ciencias Medicas, Universidade Estadual de Campinas, Campinas, SP, BR; II Departamento de Medicina, Universidade Federal de Sao Carlos, Sao Carlos, SP, BR

**Keywords:** Post-Traumatic Stress Disorder, Maternal Morbidity, Maternal Near Miss

## Abstract

**OBJECTIVE:**

To evaluate the occurrence of Post-Traumatic Stress Disorder among women experiencing a severe maternal morbidity event and associated factors in comparison with those without maternal morbidity.

**METHODS:**

In a retrospective cohort study, 803 women with or without severe maternal morbidity were evaluated at 6 months to 5 years postpartum for the presence of Post-Traumatic Stress Disorder. Interviews were conducted by telephone and electronic data was stored. Data analysis was carried out by using χ^2^, Fisher’s Exact test, and logistic regression analysis.

**RESULTS:**

There was no significant change in the prevalence of Post-Traumatic Stress Disorder related to a previous severe maternal morbidity experience. There were also no differences in diagnostic criteria for severe maternal morbidity (hypertensive syndromes, hemorrhage, surgical intervention or intensive care unit admission required, among other management criteria). Low parity (2.5-fold risk) and increasing age were factors associated with Post-Traumatic Stress Disorder.

**CONCLUSIONS:**

A severe maternal morbidity episode is not associated with Post-Traumatic Stress Disorder symptoms within five years of the severe maternal morbidity event and birth. However, a more advanced maternal age and primiparity increased the risk of Post-Traumatic Stress Disorder. This does not imply that women who had experienced a severe maternal morbidity event did not suffer or need differentiated care.

## INTRODUCTION

Severe maternal morbidity (SMM) and maternal near miss (MNM) have increasingly been referred to as important markers of maternal health and a resource to evaluate the quality of obstetric care 1,2. A continuum of morbidity exists between a healthy pregnancy and maternal death; survivors of the conditions that may lead to death are considered MNM cases 2.

Pregnancy and childbirth, even without complications, are potentially stressful situations and women may experience depression for prolonged periods after childbirth 3. Unplanned pregnancies, a negative subjective experience during childbirth, fear of maternal or infant death, limited support offered by the health team were associated with a higher prevalence of post-traumatic stress disorder (PTSD) 4,5.

Women experiencing obstetric complications have a higher risk of developing clinical and mental health problems 6,7. Similar to their children, they are more physically and socially vulnerable, and have higher risk of death up to one year postpartum 8. Less severe but also important repercussions, including decrease in functioning 9, delay in resuming sexual intercourse 10, and deterioration in quality of life may occur in association with SMM 11. Feelings such as fear of death, hopelessness and loss of female identity have been identified in other studies 12,13.

Post-traumatic stress disorder (PTSD) is associated with higher levels of physical, social and work-related impairment, leading to higher health service use and increased medical costs 14. During pregnancy and the postpartum period, the disorder may have a major impact, since maternal mental health issues may negatively interfere in infant cognitive and language development 15 and may affect the partner and family dynamics 6.

PTSD is defined as a syndrome that can be triggered after exposure to a traumatic event involving death or a consistent threat of death or severe damage to the self or others (criterion A), which elicits a subjective response of intense fear, helplessness or impotence in the midst of the situation and horror. In response to such a traumatic event, a triad of symptoms characterizes the disorder: reliving the traumatic event (criterion B), avoiding stimuli associated with trauma (Criterion C), and having symptoms of increased psychic excitability (irritability, difficulty sleeping and concentrating) 14.

Severe preeeclampsia and SMM were recently identified as a risk factors for PTSD 6,16-18. Therefore, understanding which disorders affect women after a SMM episode and the repercussions on their mental health is a key-point. The purpose of this study was to identify the prevalence of PTSD after an SMM event and the factors associated with this diagnosis.

## METHODS

This is an analysis of a retropective cohort study investigating a SMM group and a control group without any maternal complication. Details on the method of this study have already been published elsewhere 19. Briefly, its general purpose was to evaluate several aspects of the woman’s life following a SMM episode: general and reproductive health conditions, quality of life, sexual activity, capabilities and functioning, mental health, and growth and development of the child born in that pregnancy. It was conducted in a tertiary referral maternity hospital in Campinas, in the southeast of Brazil.

The SMM group was selected from women admitted to intensive care unit (ICU) during pregnancy that met the criteria for Potentially Life-Threatening Conditions (PLTC) or Maternal Near Miss (MNM) as recommended by the WHO 2, generally recognized as Severe Maternal Morbidity. The control group was composed of women with a full-term birth without any severe complications. Each control was randomly selected on the same year of birth as SMM cases to maintain a balance between groups regarding time after birth. All women who fulfilled the above requirements and had given birth from 2008 to 2012 were eligible for the study. The period between childbirth and the interview ranged from 6 months to 5 years. Data collection was conducted by chart review to identify criteria for PLTC and MNM, birth and hospital discharge conditions; and by telephone interview applying instruments to evaluate quality of life and PTSD. Information for PTSD assessment have already been collected and validated through telephone interviews 20.

From electronic clinical records, 1,157 women fulfilling selection criteria were identified. A team trained in teleresearch, using standardized CATI (computer-assisted telephone interview) procedures, searched for these women. Several phone calls were done at different times, and 840 eligible women were successfully traced and invited to participate in the study. Women agreeing to participate recorded their consent and were then interviewed by telephone, or were scheduled for interview at a more opportune date and hour. The interview was recorded and data immediately inserted into the electronic Lime Survey^®^ database. For quality control, a teleresearcher supervisor randomly selected a sample fraction of about 5% of cases and listened to the recording, checking responses marked in the electronic database. Corrections were made when necessary, and any possible errors found were reported to the interviewer.

Sample size was determined by previous studies and included all the outcome variables and was considered to have enough power to assess differences in quality of life. Presuming that 50% of women had severe physical and emotional problems during the first year following delivery, an absolute difference of 11% between both groups, a Type I error of 5% and a Type II error of 10%, each group would require 337 women, totaling 674.

PTSD was evaluated by an instrument, the PTSD Checklist-Civilian Version (PCL-C) 21, already adapted and validated in Brazil for Portuguese language 22. The current study is not a validation of the content or psychometric properties of this instrument, but only its application in a sample population of women in the postpartum period. It was composed of 17 questions with a severity scale ranging from 1 to 5 (nothing to a lot). To make the diagnosis, 4 types of symptoms are required: Criterion A (triggering condition), Criterion B (questions 1 to 5), Criterion C (questions 6 to 12) and Criterion D (questions 13 to 17). Childbirth may be considered a stressful event in itself, including the experience of pregnancy and stress of labor.

For instrument analysis, three different criteria of analysis were used. For cluster analysis, PTSD diagnostic criteria were considered according to the DSM-IV: having a clinically significant symptom from group B, having three symptoms from group C and two from group D. To be considered clinically significant, a symptom received a score that should be equal to or greater than three. For remaining analyses, a total score was used and cutoff points were defined (40 and 50) 23 that refer to the sum of scores in each question of the instrument, with values ranging from a minimum of 17 to a maximum of 85 points. The higher the score, the worse was the response to PTSD.

Data was submitted to strict checking procedures for completion and internal consistency. Missing and/or incorrect data were confirmed in recordings, medical charts or alternatively by a repeat phone call to the woman, and then corrected in the database.

For statistical analysis, sociodemographic, pregnancy and childbirth characteristics were compared between groups. The prevalence of PTSD was then determined using the 3 different sets of criteria (cluster, cutoff ≥40 and ≥50) for both groups with differences assessed using Chi-square or Fisher’s Exact tests. For women diagnosed with PTSD, the distribution between those with or without SMM and according to sociodemographics and pregnancy characteristics was provided for the three criteria. In addition, prevalences of PTSD between groups and according to the time since delivery were reported for all three sets of criteria, and according to diagnostic criteria used for SMM. Finally, multinomial regression analysis was performed to identify conditions independently associated with PTSD. For these procedures, the SPSS package was used (version 20; IBM, Armonk, NY, USA). The study was approved by the local Institutional Review Board (letter of approval CEP 233/2009). All participants recorded an audio consent form and/or signed a written version of the consent. The principles of the Helsinki Declaration of 1975, which was revised in 1983, were followed.

## RESULTS

**Figure 1:**
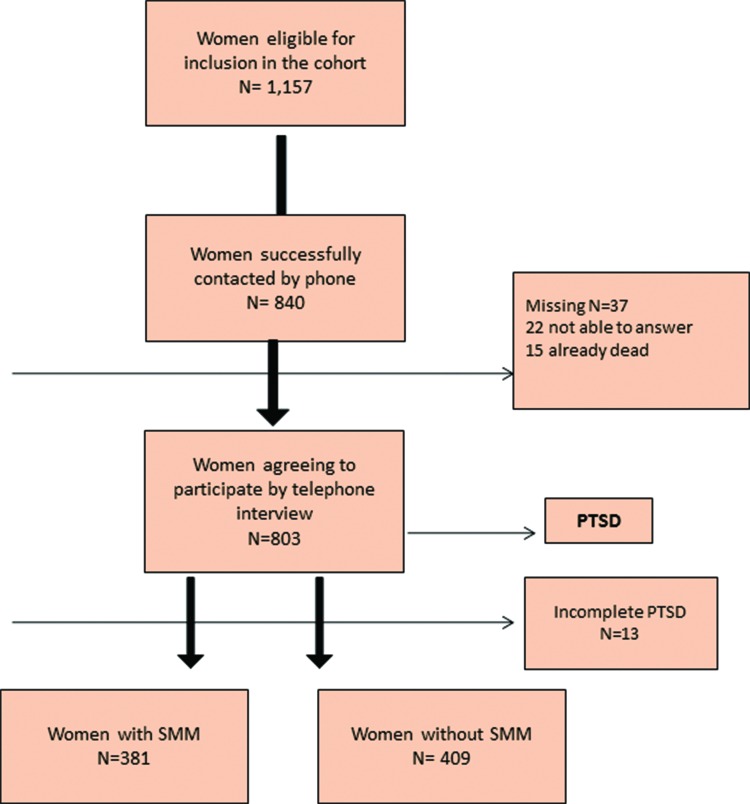
Flow chart of women in the study inclusion. PTSD: post-traumatic stress disorder; SMM: severe maternal morbidity.

Initially 1,157 women were eligible for the study, 840 of them were traced by telephone and 803 responded to the PCL-C instrument for PTSD assessment. Among the 37 missing cases, 22 did not respond the questionnaire due to diverse personal reasons and 15 had already died in different periods after childbirth (9 from late maternal causes). The tracing rate was 72.6% and the response rate was 69.4%, although 13 women did not fully complete the questionnaire. Among 790 women with complete interviews, 381 had experienced an SMM episode, while 409 had no severe complications and served as the control group (Figure 1).

The majority of women with SMM were older and more than 35% were over 35, as shown in Table 1. Groups did not show diferences in parity, ethnicity, school education, socioeconomic status, time since childbirth, having a partner and neonatal outcomes. The proportion of women with SMM undergoing cesarean section was very high (82%) in comparison with controls (48%). Briefly, women with SMM were proportionally older and had higher parity.

Table 2 shows the prevalence of PTSD using distinct criteria. By the method for cluster symptom analysis, 54.3% of women with SMM and 51.8% of those without SMM developed PTSD. In addition, no significant differences were found between groups when using the cut-off point ≥40 for subsyndromic PTSD, or a cutoff ≥50.

Table 3 shows only women diagnosed with PTSD by the three different sets of criteria comparatively with and without SMM and according to sociodemographics and pregnancy characteristics. Among women with PTSD, maternal age ≥35 years was more common in the SMM group, i.e. older women had SMM more frequently. A significantly higher proportion of women with PTSD by the cutoff ≥50 had given birth within 2 years of the interview in the SMM group. The proportion was significantly higher in more than 80% women with SMM and PTSD, than in women without SMM. For the remainig variables, there were no significant differences between groups.

Table 4 reports that there was no difference in the prevalence of PTSD in women with and without SMM at any time periods considered. The prevalence of PTSD showed no statistically significant differences according to the major determinants of SMM (hypertensiion, hemorrhage, surgical intervention or ICU admission). Therefore, the presence of a specific SMM condition did not modify the prevalence of PTSD in this sample (Data not shown).

In Table 5, the variables significantly associated with diagnosis of PTSD by a cutoff ≥50 were primiparity and maternal age above 40 years. Women with only one child had a 2.5 times higher risk of having PTSD than multiparous women. In contrast, in women aged 20 to 39 the risk of PTSD decreased by 50 to 60%. The presence of SMM was not independently associated with the risk of PTSD.

## DISCUSSION

The current study did not find an association between SMM and PTSD in this population, at least within one to 5 years of the SMM event. This does not mean that women, who had a SMM episode but no signs of PTSD, did not suffer any traumatic experience. The evaluation of PTSD at different times in the postpartum period showed that a higher proportion of women with a SMM episode were older and underwent cesarian section more frequently, which was probably related to the occurrence of maternal morbidity.

The association between advanced maternal age and SMM, as well as increased maternal age and higher likelihood of cesarean section, have already been described. Pregnancies at a later maternal age are more likely to have complications, mainly due to maternal premorbid conditions, as hypertension and diabetes 24, what may partly explain our results. However, even when indicated for maternal and fetal conditions, cesarean section is not a risk-free intervention. It can be very difficult to distinguish between cause and effect in the relationship between cesarean delivery and maternal morbidity 25.

The prevalence of PTSD was no different between morbidity groups using any criteria of analysis. Therefore, in this sample, the occurrence of PTSD was not influenced by SMM. These results differ from a study that described preeclampsia as an important factor predisposing to PTSD 17. Another cohort study evaluating British women at 6 to 8 weeks postpartum also identified a higher risk of PTSD symptoms among those experiencing severe maternal morbidity 18.

On the other hand, cesarean delivery appeared to be strongly associated with SMM, regardless the presence of PTSD with any criteria. This could probably be due to the impact of a potential threat to maternal or neonatal life during childbirth. It is not the birth event itself that may lead a woman to trauma, but her perception of this event. A birth may seem perfect and properly cared by the professional, but it is not necessarily felt the same way by the woman. In addition to performing a technically suitable procedure, it is vital to consider the issues that extend beyond traditional clinical parameters, towards a more personal care centered on the woman herself.

In women with an SMM episode, PTSD was higher with the cutoff ≥50 within 2 years of childbirth. This was the only positive result found for time since the occurrence of the morbid episode, suggesting that stress may decrease over time.

Several factors may contribute to PTSD in the postpartum period. Subjective suffering during childbirth and obstetric emergencies were the most important risk factors for PTSD found in a recent systematic review. Furthermore, emergency cesarian section, instrumental vaginal delivery and neonatal complications were also important predictors of PTSD 26.

A meta-analysis estimated that the prevalence of PTSD was 5-fold higher in women at risk 27. The perception of a negative subjective experience of childbirth and of limited support during childbirth were also factors strongly associated with PTSD after childbirth in another meta-analysis 28. They also found a strong association between difficulties in maternal coping and postpartum depression. In our study, we had no information on women’s perception of maternal care received. However, we can infer that it may have been a more positive experience, or it was perceived no differently from women without complications, considering that SMM was not identified as an important factor for PTSD.

Estimates of PTSD after childbirth have varied enormously among studies. Many factors, including subjective elements, childbirth care models and personal history of the woman have been identified as important predictors of PTSD. Therefore, it is difficult to point out a specific risk factor for PTSD or make a single estimate of its prevalence following a SMM event.

In the current study, it is noteworthy that women were evaluated within 5 years of the event. This may be a positive differential in comparison to other studies on PTSD after childbirth. Even after stratifying women by time between childbirth and the interview, in this study no association between SMM and PTSD was observed. However, the study has not enough power to identify difference between groups according to time since the morbidity event, which could be a study limitation. Additional studies are recommended for the investigation of potential short-term and long-term effects of SMM on mental health of women and their families, preferably with multicenter studies with a wider population-based representativeness.

Our results indicate that a severe maternal morbidity experience is not more significantly associated with PTSD symptoms within five years of the SMM event. Findings did not imply that suffering did not occur or that differentiated and integrated care is not required in this group of women.

## AUTHOR CONTRIBUTIONS

Angelini CR, Cecatti JG, Pacagnella RC, Parpinelli MA and Andreucci CB conceived and designed the experiments. Angelini CR, Andreucci CB, Pacagnella RC, Silveira C, Parpinelli MA, Ferreira EC, Santos JP, Zanardi DM and Cecatti JG performed the experiments. Angelini CR, Pacagnella RC and Cecatti JG analyzed the data. Angelini CR wrote the paper. Angelini CR, Andreucci CB, Pacagnella RC, Silveira C, Parpinelli MA, Ferreira EC, Zanardi DM, Santos JP, Souza RT and Cecatti JG reviewed, provided suggestions and agreed on the final version of the manuscript.

## Figures and Tables

**Table 1:** Sociodemographics and pregnancy characteristics of women according to a previous experience of severe maternal morbidity (SMM).

Sociodemographics and pregnancy characteristics	With SMM		Without SMM		*p*-value*
	n	%	n	%	
**Maternal age (years)**					**0.001**
<20	16	4.2	24	5.9	
20 a 24	58	15.2	80	19.6	
25 a 29	86	22.6	110	26.9	
30 a 34	86	22.6	108	26.4	
35 a 39	82	21.5	54	13.2	
≥40	53	13.9	33	8.1	
Total	381		409		
**Parity**					0.244
1	98	31.8	109	34.0	
2	93	30.2	103	32.1	
3	54	17.5	63	19.6	
≥4	63	20.5	46	14.3	
Total	308		321		
**Skin color/ethnicity**					0.204
White	197	51.7	193	47.2	
Non white	184	48.3	216	52.8	
Total	381		409		
**Literacy (years)**					0.238
Up to 4	24	6.7	14	3.6	
5-8	103	28.9	102	26.4	
9-11	194	54.5	233	60.4	
≥12	35	9.8	37	9.6	
Total	356		386		
**Socioeconomic classification**					0.193
Class A+B	117	38.6	140	43.8	
Class C+D+E	186	61.4	180	56.3	
Total	303		320		
**Time since delivery (y)**					0.302
1 - 2	152	39.9	178	43.5	
3 - 5	229	60.1	231	56.5	
Total	381		409		
**With a partner**					0.926
Yes	255	82.2	265	83.1	
No	53	17.2	54	16.9	
Total	308		319		
**Mode of delivery**					**<0.001**
Vaginal	67	18.0	211	51.6	
Cesarean section	305	82.0	198	48.4	
Total	372		409		
**Neonatal outcome**					0.054
Alive	299	95.8	391	98.2	
Death	13	4.2	7	1.8	
Total	312		398		

* χ^2^ test.

**Table 2:** Prevalence of PTSD among women according to a previous experience of severe maternal morbidity (SMM) using three different criteria for analysis of the PCL-C instrument.

Criteria for PTSD	With SMM		Without SMM		*p*-value*
**Criteria for Cluster**	**n**	**%**	**n**	**%**	0.482
Positive	207	54.3	212	51.8	
Negative	174	45.7	197	48.2	
**Cut-off ≥40**	**n**	**%**	**n**	**%**	0.183
Positive	196	51.4	191	49.4	
Negative	185	48.6	218	54.1	
**Cut-off ≥50**	**n**	**%**	**n**	**%**	0.479
Positive	113	29.7	112	27.4	
Negative	268	70.3	297	72.6	
Total	381		409		

* χ^2^ test.

**Table 3:** Women diagnosed with PTSD with or without severe maternal morbidity (SMM) using 3 different sets of criteria for the PCL-C instrument according to some sociodemographic and pregnancy characteristics.

Sociodemographic and pregnancy characteristics	Criteria for Cluster		Cut-off ≥40		Cut-off ≥50	
	With SMM %	Without SMM %	With SMM %	Without SMM %	With SMM %	Without SMM %
**Maternal age (years)**	n=419		n=387		n=225	
<20	3.9	7.5	4.6	6.8	2.7	7.1
20 a 24	15.9	19.8	15.8	19.4	12.4	19.6
25 a 29	24.2	26.9	21.4	27.2	25.7	24.1
30 a 34	22.2	27.8	23.0	28.3	22.1	30.4
35 a 39	21.3	10.8	21.4	11.5	26.5	12.5
≥40	12.6	7.1	13.8	6.8	10.6	6.3
*p*-value	**0.009**		**0.014**		**0.027**	
**Parity**	n=348		n=324		n=191	
1	31.0	31.6	30.2	30.9	26.9	28.4
2	24.6	33.3	24.1	32.1	26.0	36.8
3	18.7	19.8	19.1	21.0	24.0	16.8
≥4	25.7	15.3	26.5	16.0	28.1	17.9
*p*-value	0.070		0.103		0.115	
**Skin color/ethnicity**	n=419		n=387		n=225	
White	46.9	45.3	48.0	46.1	46.0	46.4
Non white	53.1	54.7	52.0	53.9	54.0	53.6
*p*-value	0.746		0.710		0.951	
**Literacy (years)**	n=402		n=373		n=217	
Up to 4	5.6	3.9	5.9	4.3	7.5	3.6
5-8	32.1	26.7	33.5	26.1	31.8	30.0
9-11	55.1	62.6	53.5	61.7	52.3	58.2
≥12	7.1	6.8	7.0	8.0	8.4	8.2
*p*-value	0.452		0.326		0.606	
**Socioeconomic classification**	n=303		n=322		n=190	
Class A+B	34.9	40.7	33.8	40.1	32.6	38.9
Class C+D+E	65.1	59.3	66.3	59.9	67.4	61.1
*p*-value	0.269		0.236		0.364	
**Time since delivery (y)**	n=381		n=387		n=225	
1 - 2	58.5	54.7	58.7	55.0	53.3	46.4
3 - 5	41.5	45.3	41.3	45.0	46.7	53.6
*p*-value	0.440		0.463		**0.039**	
**With a partner**	n=308		n=323		n=191	
Yes	78.4	83.5	79.0	82.0	76.0	81.1
No	21.6	16.5	21.0	18.0	24.0	18.9
*p*-value	0.221		0.500		0.399	
**Mode of delivery**	n=414		n=382		n=222	
Vaginal	15.9	51.9	16.2	51.3	18.2	58.9
Cesarean section	84.2	48.1	83.8	48.7	81.8	41.1
*p*-value	**0.000**		**0.000**		**0.000**	
**Neonatal outcome**	n=379		n=346		n=204	
Alive	94.7	97.6	94.3	97.3	94.7	96.4
Death	5.3	2.4	5.7	2.7	5.3	3.6
*p*-value*	0.142		0.153		0.560	

* χ^2^ test.

**Table 4:** Prevalence of PTSD among women with or without severe maternal morbidity (SMM) according to criteria used for analysis of the PCL-C instrument stratified by time since delivery until interview.

Time since delivery	With SMM		Without SMM		*p*-values*
	n	%	n	%	
**5 years (2008)**					
Criteria for cluster	11	37.9	13	41.9	0.752
Cut-off ≥40	11	37.9	13	41.9	0.752
Cut-off ≥50	7	24.1	4	12.9	0.261
**4 years (2009)**					
Criteria for cluster	59	53.2	47	50.0	0.653
Cut-off ≥40 *	55	49.5	41	43.6	0.404
Cut-off ≥50 *	31	27.9	21	22.3	0.421
**3 years (2010)**					
Criteria for cluster	51	57.3	56	52.8	0.532
Cut-off ≥40	49	55.1	51	48.1	0.334
Cut-off ≥50	30	33.7	27	25.5	0.208
**2 years (2011)**					
Criteria for cluster	51	57.3	72	58.5	0.857
Cut-off ≥40	46	51.7	63	51.2	0.947
Cut-off ≥50	26	29.2	47	38.2	0.174
**1 year (2012)**					
Criteria for cluster	35	55.6%	24	43.6%	0.196
Cut-off ≥40	35	55.6%	23	41.8%	0.136
Cut-off ≥50	19	30.2%	13	23.6%	0.427

* χ^2^ or Fisher’s Exact test.

**Table 5:** Factors independently associated with the diagnosis of PTSD using a cutoff point ≥50 as a criterion in a multivariate regression analysis.

Predictors	OR	95% CI		*p*-value
**Maternal age**				
<20	0.483	0.157	1.484	0.204
20 a 24	**0.376**	**0.170**	**0.832**	**0.016**
25 a 29	**0.448**	**0.219**	**0.918**	**0.028**
30 a 34	**0.461**	**0.228**	**0.930**	**0.030**
35 a 39	**0.428**	**0.208**	**0.882**	**0.021**
≥40	Ref.			
**Parity**				
1	**2.553**	**1.408**	**4.629**	**0.002**
2	1.636	0.956	2.799	0.072
3	1.335	0.754	2.365	0.322
≥4	Ref.			
**Ethnicity**				
White	1.085	0.758	1.555	0.655
Non white	Ref.			
**Schooling (years)**				
Up to 4	0.931	0.331	2.619	0.892
5-8	1.033	0.497	2.150	0.930
9-11	0.969	0.519	1.810	0.922
≥12	Ref.			
**Socioeconomic classification**				
Class A+B	1.215	0.809	1.825	0.348
Class C+D+E	Ref.			
**With a partner**				
Yes	1.490	0.945	2.349	0.086
No	Ref.			
**Maternal morbidity**				
Without SMM	1.094	0.766	1.564	0.621
With SMM	Ref.			
